# Risk stratification for stricture formation after endoscopic submucosal dissection for esophageal dysplasia

**DOI:** 10.1093/dote/doaf096

**Published:** 2025-11-03

**Authors:** Kareem Khalaf, Youstina Hanna, Tomoyuki Nishimura, Huaqi Li, Natalia Causada Calo, Gary R May, Christopher W Teshima, Jeffrey D Mosko

**Affiliations:** Division of Gastroenterology, St. Michael’s Hospital, University of Toronto, Toronto, ON, Canada; Division of Gastroenterology, St. Michael’s Hospital, University of Toronto, Toronto, ON, Canada; Division of Gastroenterology, St. Michael’s Hospital, University of Toronto, Toronto, ON, Canada; Division of Gastroenterology, St. Michael’s Hospital, University of Toronto, Toronto, ON, Canada; Division of Gastroenterology, St. Michael’s Hospital, University of Toronto, Toronto, ON, Canada; Division of Gastroenterology, St. Michael’s Hospital, University of Toronto, Toronto, ON, Canada; Division of Gastroenterology, St. Michael’s Hospital, University of Toronto, Toronto, ON, Canada; Division of Gastroenterology, St. Michael’s Hospital, University of Toronto, Toronto, ON, Canada

**Keywords:** Barrett’s esophagus, endoscopic submucosal dissection, esophageal cancer, esophageal stricture, squamous cell carcinoma

## Abstract

We aimed to evaluate the demographic, clinical, procedural, and histopathologic factors associated with stricture development following esophageal endoscopic submucosal dissection (ESD). We conducted a retrospective cohort study of patients undergoing ESD for esophageal lesions from 2019 to 2024 at St. Michael’s Hospital, in Toronto, Canada. The primary outcome was stricture formation, defined as a symptomatic luminal narrowing at the ESD site confirmed on follow-up endoscopy, requiring intervention. Strictures requiring dilation developed in 24% of patients, 85% of which were impassable with a standard gastroscope (9.9 mm diameter). Stricture rates increased with defect circumferential involvement: <50% (7.7%), 50%–74% (11.5%), 75%–89% (23.1%), and ≥90% (57.7%). Intraprocedural local triamcinolone acetate (LTA) injection was administered in 40 of 108 patients (37%), with a mean defect circumferential size of 87.5%. Among patients receiving LTA, stricture rates varied based on defect size: for <50% circumferential defect involvement (*n* = 1) and 50%–74% (*n* = 3), no strictures developed; for 75%–90% (*n* = 17), 6 patients (35%) developed strictures, 5 of which were impassable; and for 90%–100% (*n* = 19), 11 patients (58%) developed strictures, all of which were impassable. Patients selectively discharged on prophylactic steroids demonstrated varied stricture rates depending on the steroid regimen: prednisone (61.5%), oral budesonide (26.9%), and combination therapy (7.7%). Independent predictors of stricture formation included defect circumferential involvement (OR 1.07, 95% CI 1.03–1.12, *p* < 0.001), length of hospitalization (OR 1.88, 95% CI 1.11–3.16, *p* = 0.018), and presence of deep mural injury (OR 6.28, 95% CI 1.10–35.88, *p* = 0.039). Stricture formation post-ESD is strongly associated with lesion and procedural characteristics, including defect circumferential involvement, deep mural injury, and length of hospitalization.

## INTRODUCTION

Endoscopic submucosal dissection (ESD) has emerged as a preferred technique for the *en bloc* resection of early esophageal neoplasia, including squamous cell dysplasia (SCD) and Barrett’s esophagus (BE)–related dysplasia.^1–3^ Compared to endoscopic mucosal resection, ESD enables wider, more extensive resections, reducing local recurrence rates.^4^ However, post-ESD esophageal stricture formation remains a significant complication, leading to dysphagia, morbidity, and the need for repeated endoscopic interventions.^5^^,^^6^ Stricture risk is multifactorial, influenced by both lesion characteristics and procedural factors. The extent of circumferential mucosal resection is the most well-established predictor, with larger defects carrying a higher risk of stricture.^7^ Other potential contributors include deep mural injury (DMI), prolonged procedure duration, and post-resection inflammation.^8^ Despite efforts to mitigate strictures using local steroid injections, systemic steroids, and alternative adjunctive measures, no standardized prophylactic approach has demonstrated universal efficacy.^9^^,^^10^ Given the significant morbidity associated with post-ESD strictures, identifying key risk factors is essential to optimize patient management. While prior studies have explored predictors of stricture formation, much of the existing literature focuses on individual interventions rather than comprehensive risk stratification.^11^^,^^12^ A detailed evaluation of lesion characteristics, procedural factors, and post-procedural management strategies may provide valuable insights into stricture risk and guide clinical decision-making. Reported post-ESD esophageal stricture rates vary by region, pathology, and circumferential extent. Western Barrett’s-focused ESD cohorts with limited resections report low overall stricture rates (e.g. 2.1% in a three-center study).^13^ By contrast, Eastern squamous-predominant series show high rates when defects are ≥3/4 to circumferential without robust prophylaxis.^14^ This study reflects a Western, tertiary-center case-mix enriched for Barrett’s-related neoplasia. By characterizing independent risk factors of stricture formation, the study aims to facilitate a more tailored approach to post-ESD care. An improved understanding of these factors may allow for targeted prophylactic strategies, including the judicious use of steroids or other adjunctive therapies, to reduce the burden of post-ESD stenosis and ultimately improve patient outcomes.

## METHODS

### Study design and population

This single-center retrospective cohort study was conducted at St. Michael’s Hospital, Toronto, Canada. The study evaluated patients who underwent ESD for esophageal dysplasia between January 1, 2019, and November 30, 2024. Institutional review board approval was obtained (21-083), and informed consent was waived due to the retrospective nature of the study. The study adhered to the Strengthening the Reporting of Observational Studies in Epidemiology (STROBE) guidelines,^15^ details can be found in Supplementary Data—Table S1. This study included adult patients (≥18 years) with histologically confirmed BE–related or squamous cell dysplasia undergoing endoscopic submucosal dissection. Patients were excluded if they underwent esophagectomy following adverse pathology or were lost to follow-up, with no follow-up endoscopy to assess the resection site. Follow-up involved clinical evaluations and surveillance endoscopy to document outcomes, including stricture formation. This process was designed to minimize selection bias and ensure that only relevant cases contributed to the study findings.

### Endoscopic procedure method

All ESD procedures were performed by an experienced endoscopist with expertise in advanced therapeutic endoscopy (GM, JM, CT). Procedures were conducted under general anesthesia. Prior to the procedure, all patients underwent a detailed endoscopic evaluation with high-definition white-light endoscopy (WLE) and image-enhanced endoscopy, including narrow-band imaging (NBI) or blue-light imaging (BLI), to characterize lesion morphology, predicted histology, and suspected depth of invasion. Endoscopic ultrasound (EUS) was selectively performed for lesions with suspected deep submucosal invasion to rule out muscularis propria involvement. ESD was performed using a high-definition gastroscope with a water jet system. Carbon dioxide insufflation was used in all cases to minimize procedural complications. Lesion margins were delineated with diathermic markings using the ESD knife. A submucosal lifting solution (Voluven or normal saline) with methylene blue was injected to facilitate safe dissection. ESD devices and approach were at the discretion of the proceduralist. Intraprocedural bleeding was managed with hemostatic forceps (Coagrasper) or the electrosurgical knife. If a DMI occurred, clip closure was performed. In cases with extensive circumferential defects (≥75% of the luminal circumference), prophylactic steroid injections (LTA) were administered to reduce post-ESD fibrosis. LTA was injected in a diluted solution (40 mg diluted in 100 mL saline) at multiple points (0.5–1 mL per site) along the resection margin to prevent cicatricial contraction. However, in cases with DMI, steroid injection was avoided in those areas due to concerns about muscle atrophy. Selected patients were discharged on a regimen of systemic steroids (prednisone or budesonide or combination) based on resection size. Patients were monitored in the hospital post-procedure for immediate complications and maintained on a liquid diet for the first 24–48 hours. A high-dose intravenous proton pump inhibitor (PPI) regimen was initiated to promote mucosal healing. Follow-up endoscopy timing was based on stricture risk assessment. In patients with large circumferential defects (>75%), follow-up endoscopy was typically performed within 4–8 weeks to monitor for stricture formation and initiate prophylactic dilation if necessary. For patients with reassuring pathology and a lower risk of stricture, follow-up endoscopy was scheduled at 3–6 months.

### Expected outcomes and definitions

The primary outcome was to identify risk factors and independent predictors of stricture formation, defined as symptomatic luminal narrowing at the ESD site requiring intervention (endoscopic dilation), confirmed on follow-up endoscopy. Symptomatic stricture was defined as the presence of dysphagia or food impaction related to the ESD site. Strictures were categorized based on severity, determined by gastroscope passage, with severe strictures defined as symptomatic narrowing impassable with a standard gastroscope (mean 9.9 mm diameter). Secondary outcomes included evaluating the efficacy of prophylactic therapies such as local triamcinolone injection and systemic steroids.

### Statistical analysis

Descriptive statistics were used to summarize patient characteristics, treatment modalities, complications, and long-term outcomes. Continuous variables were reported as means and interquartile ranges (IQRs), depending on their distribution. Categorical variables were expressed as frequencies and percentages. Independent-samples *t*-tests and chi-square tests were used appropriate for the data. We conducted multivariate logistic regression with backward stepwise entry using the likelihood ratio to determine the impact of variables on stricture formation. We constructed models to predict the primary outcome and the secondary outcome. We selected predictor variables for the multivariable model based on clinical expertise and existing literature. Baseline patient characteristics included demographic factors (age, sex), significant variables identified from univariate analysis, lesion location, defect circumferential involvement, predicted histology, prior treatments, intraprocedural complications, invasion depth, and prophylactic treatments. Variance and multicollinearity were assessed during model development to ensure the stability and reliability of estimates. Variance inflation factors (VIFs) were calculated, with predictors exhibiting a VIF >5 excluded from the final multivariate model. Variables were retained in the final model only if they remained significantly associated with the primary outcome in multivariate logistic regression analysis. For effect size measures, the odds ratio (OR) with 95% confidence interval was used for Fisher’s exact test and Cohen’s d was used for the independent-samples *t*-test, respectively. Statistical analyses were performed using R software (version RStudio 2024 .04.2 Build 764). A *p*-value of <0.05 was considered statistically significant.

## RESULTS

**Fig. 1:**
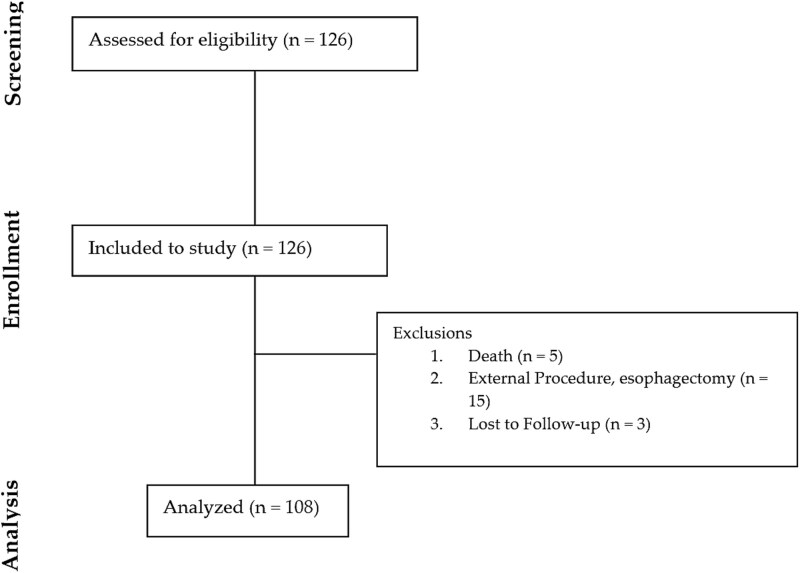
Study flow chart.

A total of 126 patients underwent ESD for esophageal lesions between 2019 and 2024. Of these, 18 patients were excluded from follow-up due to adverse pathology requiring esophagectomy (*n* = 15) or loss to follow-up (*n* = 3), leaving 108 patients for analysis (Fig. 1). A total of 26 of the 108 patients (24.1%) developed strictures post-ESD requiring dilation (Table 1). Of these 26 patients, 22 (84.6%) experienced strictures impassable with standard endoscopes. Overall, 24 patients (92.3%) required balloon dilation as treatment for their strictures. Notably, only 16 (61.5%) of these patients had a prior diagnosis of BE, while the remainder developed strictures in the setting of squamous cell dysplasia; balloon dilation was necessary for nearly all strictures, independent of the underlying pathology. Demographic variables including age, sex, comorbidities, medications, and prior endoscopic procedures were similar between patients with and without stricture formation post-ESD. Of the patients who developed strictures, 24 (92.3%) had BE and 2 (7.7%) had SCD. Compared to patients without strictures, a smaller proportion of those with strictures had a prior diagnosis of BE (61.5% vs. 84.2%, *p* = 0.014). There were fewer patients with prior gastrointestinal surgeries who developed strictures (11.5% vs. 31.7%, *p* = 0.043). There were, however, differences in the clinical disease characteristics between the two groups on univariate analysis. Patients who developed strictures overall had larger lesion sizes (lesion size ≥5 cm: 46.2% vs. 19.5%, *p* = 0.051) and occupied significantly more luminal circumference (90%–100%: 26.9% vs. 11.0%, *p* = 0.015) compared to patients who did not develop strictures. Otherwise, there were no significant differences in lesion location, Paris classification, or high-risk features (submucosal invasion depth, poor differentiation, and lymphovascular invasion) between patients who did and did not develop strictures post-ESD.

**Table 1:** Demographic and disease characteristics in patients with and without stricture formation

	**Stricture *n* = 26**	**No stricture *n* = 82**	***p*-value** ^*^
**Age, mean (range)**	69 (55–88)	68 (34–87)	0.395
**Sex, male *n* (%)**	22 (84.6%)	68 (82.9%)	0.841
**Comorbidities *n* (%)**			
**Cardiovascular**†	16 (61.5%)	52 (63.4%)	0.863
** Respiratory** **‡**	5 (19.2%)	11 (13.4%)	0.467
** Diabetes**	6 (23.1%)	14 (17.1%)	0.492
** Malignancy**	1 (3.9%)	8 (9.8%)	0.342
**GI diseases *n* (%)**			
** Previously noted BE**	16 (61.5%)	69 (84.2%)	0.014
** GERD**	9 (34.6%)	27 (32.9%)	0.874
** Achalasia**	0 (0.0%)	1 (1.2%)	0.572
** IBD** §	0 (0.0%)	4 (4.9%)	0.251
**Prior GI surgery n (%)**	3 (11.5%)	26 (31.7%)	0.043
**Medications n (%)**			
** Antithrombotic**	7 (26.9%)	20 (24.4%)	0.795
** PPI**	16 (61.5%)	48 (58.5%)	0.786
** Steroid**	0 (0.0%)	1 (1.2%)	0.572
** Chemotherapy**	0 (0.0%)	1 (1.2%)	0.572
**Dysplasia type *n* (%)**			0.103
** SCC**	2 (7.7%)	18 (22.0%)	
** BE**	24 (92.3%)	64 (78.1%)	
**Prior endoscopic procedures *n* (%)**			
** EMR**	3 (11.5%)	7 (8.5%)	0.645
** RFA**	2 (7.7%)	2 (2.4%)	0.217
**Lesion size *n* (%)**			0.051
** <2 cm**	1 (3.9%)	6 (7.3%)	
** 2 cm**	4 (15.4%)	25 (30.5%)	
** 2–5 cm**	9 (34.6%)	35 (42.7%)	
** ≥5 cm**	12 (46.2%)	16 (19.5%)	
**Circumferential lesion *n* %**			0.015
** <50%**	6 (23.1%)	45 (54.9%)	
** 50–74%**	8 (30.8%)	22 (26.8%)	
** 75–89%**	5 (19.2%)	6 (7.3%)	
** 90–100%**	7 (26.9%)	9 (11.0%)	
**Location *n* (%)**			0.078
** Mid esophagus**	6 (23.1%)	8 (9.8%)	
** Distal esophagus**	20 (76.9%)	74 (90.2%)	
**Paris classification *n* (%)**			0.354
** 0-Isp**	0 (0.0%)	1 (1.2%)	
** 0-Is**	1 (3.9%)	12 (14.6%)	
** 0-IIa**	14 (53.9%)	26 (31.7%)	
** 0-IIb**	5 (19.2%)	12 (14.6%)	
** 0-IIc**	1 (3.9%)	5 (6.1%)	
** 0-IIa + Is**	1 (3.9%)	9 (11.0%)	
** 0-IIa + Ic**	4 (15.4%)	17 (20.7%)	
**High-risk features**			
** Submucosal invasion depth, mm mean (range)**	0.20 (0–4.5)	0.20 (0–5.3)	0.373
** Poor differentiation**	1 (3.9%)	4 (4.9%)	0.827
** Lymphovascular invasion**	3 (11.5%)	0 (0.0%)	0.356
**Time to follow-up endoscopy, days**	58 (12–174)	133 (1–839)	<0.0001

^*^ *T*-test used for continuous variables and chi-square used for categorical variables.

^†^Cardiovascular comorbidities include CAD, CHF, HTN, DLD, PAD, valve, and arrythmia.

^‡^Respiratory comorbidities include asthma, COPD, and ILD.

§ IBD includes UC and Crohn disease.

All procedures were technically successful, resulting in en bloc resection of the target esophageal lesions. However, procedure time differed significantly between patients who did and did not develop strictures, with the former having longer procedural times (mean: 170 vs. 142 minutes, *p* = 0.010; Table 2). Intraprocedural triamcinolone injections were more common in patients who developed strictures (65.4% vs. 28.1%, *p* = 0.0006). Circumferential defect involvement was greater in patients who developed strictures compared to those who did not (90%–100% circumferential defect: 57.7% vs. 17.1%, 75%–89% circumferential defect: 23.1% vs. 15.9%, *p* < 0.0001). Adverse events during ESD occurred more often in patients who developed strictures, including DMI (42.3% vs. 9.8%, *p* = 0.0001) and perforation (7.7% vs. 0.0%, *p* = 0.011). Of the 54 total patients who developed intraprocedural bleeding, epinephrine injection and coagulation graspers were used in 9.3% and 44.4%, respectively. A total of 91.7% of patients achieved R0 resection with no significant differences between groups.

**Table 2:** ESD procedure characteristics in patients with and without stricture formation

	**Stricture *n* = 26**	**No stricture *n* = 82**	***p*-value** ^*^
**Technique *n* (%)**			0.467
**Conventional**	20 (76.9%)	57 (69.5%)	
**Pocket**	6 (23.1%)	25 (30.5%)	
**Procedure time, minutes**	170 (75–360)	142 (33–285)	0.010
**Intraprocedural injections *n* (%)**			
** Saline**	8 (30.8%)	21 (25.6%)	0.605
** Voluven**	12 (46.2%)	55 (67.1%)	0.056
** Methylene blue**	20 (76.9%)	72 (87.8%)	0.174
** Triamcinolone**	17 (65.4%)	23 (28.1%)	0.0006
** Epinephrine**	18 (69.2%)	61 (74.4%)	0.605
**Defect size *n* (%)**			0.106
** 2 cm**	1 (3.9%)	5 (6.1%)	
** 2–5 cm**	1 (3.9%)	17 (20.7%)	
** ≥5 cm**	24 (92.3%)	60 (73.2%)	
**Circumferential defect *n* (%)**			0.0001
** <50%**	2 (7.7%)	19 (23.2%)	
** 50%–74%**	3 (11.5%)	36 (43.9%)	
** 75%–89%**	6 (23.1%)	13 (15.9%)	
** 90%–100%**	15 (57.7%)	14 (17.1%)	
**Adverse events *n* (%)**			
** Intraprocedural bleed**	14 (53.9%)	40 (48.8%)	0.653
** Deep mural injury**	11 (42.3%)	8 (9.8%)	0.0001
** Perforation**	2 (7.7%)	0 (0.0%)	0.011
**R0 resection *n* (%)**	25 (96.2%)	74 (90.2%)	0.342

^*^ *T*-test used for continuous variables and chi-square used for categorical variables.

Post-procedure hospitalization was significantly longer for patients who developed strictures compared to patients who did not develop strictures (>3 days: 76.9% vs. 34.2%, *p* = 0.0001; Table 3). Overall, there were low rates of post-procedural complications for all patients, including bleeding (3/108: 2.8%), perforation (2/108: 1.9%), and infection (2/108: 1.9%). However, there were significantly more post-procedural perforation events found in patients who had developed strictures compared to those who did not develop strictures post-ESD (7.7% vs. 0.0%, *p* = 0.011). Other post-procedural complications, including bleeding (3.9% vs. 2.4%, *p* = 0.704) and infection (0.0% vs. 2.4%, *p* = 0.422), did not show a statistically significant difference between the stricture and non-stricture groups.

**Table 3:** Post-procedural characteristics

	**Stricture *n* = 26**	**No stricture *n* = 82**	*p*-value^*^
**Length of hospitalization**			0.0001
≤3 days	6 (23.1%)	54 (65.9%)	
>3 days	20 (76.9%)	28 (34.2%)	
**Prophylactic steroids**			0.544
** None**	0 (0.0%)	6 (7.3%)	
** Prednisone**	16 (61.5%)	53 (64.6%)	
** Budesonide**	7 (26.9%)	17 (20.7%)	
** Prednisone + budesonide**	2 (7.7%)	5 (6.1%)	
** Other**	1 (3.9%)	1 (1.2%)	
**Complications *n* (%)**			
** Bleed**	1 (3.9%)	2 (2.4%)	0.704
** Perforation**	2 (7.7%)	0 (0.0%)	0.011
** Infection**	0 (0.0%)	2 (2.4%)	0.422
**Post-procedural medications *n* (%)**			
** Antibiotics**	1 (3.9%)	3 (3.7%)	0.965
** PPI**	25 (96.2%)	78 (95.1%)	0.827
** Sucralfate**	21 (80.8%)	59 (72.0%)	0.371

^*^ *T*-test used for continuous variables and chi-square used for categorical variables.

On multivariate analysis, independent predictors of stricture formation included defect circumferential involvement (OR 1.07, 95% CI 1.03–1.12, *p* < 0.001), length of hospitalization (OR 1.88, 95% CI 1.11–3.16, *p* = 0.018), and presence of intra-procedural DMI (OR 6.28, 95% CI 1.10–35.88, *p* = 0.039) (Table 4). Length of hospitalization, a reflection of procedural complexity, was longer, as expected, in patients with greater defect circumferential involvement (mean 77% ± 23% for >3 days vs. 63% ± 24% for ≤3 days, *p* = 0.001). Patients hospitalized >3 days had a higher postprocedural complication rate (19%) compared to those hospitalized ≤3 days (5%, *p* = 0.001). Finally, prophylactic steroid use was marginally protective against stricture development (OR 0.98, 95% CI 0.96–1.00, *p* = 0.05).

**Table 4:** Independent variables associated with formation of stricture post-ESD

	**OR**	**95% CI**	***p*-value**
**Circumferential defect %**	1.07	1.03–1.12	<0.001
**Length of hospitalization**	1.88	1.11–3.16	0.018
**Deep mural injury**	6.28	1.10–35.88	0.039

Secondary outcomes included evaluating the efficacy of prophylactic therapies such as LTA and systemic steroids, as well as comparisons between BE-related strictures and SCD-related strictures. Among patients who developed strictures, 20.4% had severe strictures impassable with a standard gastroscope. The efficacy of prophylactic therapies was assessed, with intraprocedural triamcinolone injections being more frequently used in patients who developed strictures compared to those who did not (65.4% vs. 28.1%, *p* = 0.0006). When comparing stricture formation between BE and SCD, strictures occurred more frequently in BE-associated dysplasia (27.3% vs. 10.0%, *p* = 0.103). The likelihood of developing severe strictures was significantly associated with greater circumferential defect involvement, with 57.7% of patients with ≥90% defect circumference requiring multiple dilation sessions.

## DISCUSSION

This study evaluated the risk factors associated with stricture formation following ESD for esophageal dysplasia. Among 108 patients analyzed, 24% developed strictures requiring dilation, with 20% experiencing severe strictures impassable with a standard gastroscope. It is important to note that the definition of stricture varies significantly across the literature, with no universally accepted criteria. For this study, we defined stricture as symptomatic luminal narrowing at the ESD site requiring endoscopic intervention, as this was considered the most clinically relevant definition—focusing on strictures that directly impacted patient management and necessitated treatment. The primary factors associated with stricture development included defect circumferential involvement, DMI, and prolonged hospitalization. Our findings elucidate the significant role of lesion and procedural characteristics in post-ESD stricture risk and highlight the need for individualized risk stratification to prevent stricture formation.

Greater circumferential defect size was a strong independent predictor of stricture formation (OR 1.07, 95% CI 1.03–1.12, *p* < 0.001), with stricture rates increasing as circumferential involvement exceeded 75%. In our cohort, patients with near-complete circumferential defects (≥90%) had a stricture rate of 57.7%, compared to only 8% in those with <50% defect involvement. This progressive increase in stricture risk with greater mucosal defect size relays the significant role of resection extent in post-ESD luminal stenosis.^16^ The pathophysiology underlying this association is well documented. Extensive mucosal defects expose a larger area of the esophageal wall to post-resection inflammation and subsequent fibrosis.^17^^,^^18^ Unlike focal injuries that can heal with localized re-epithelialization, near-circumferential defects result in a more pronounced healing response characterized by excessive collagen deposition and contracture, leading to circumferential luminal constriction.^17^^,^^19^^,^^20^ The esophagus, being a relatively narrow and compliant organ, is particularly susceptible to significant lumen compromise when a substantial portion of its mucosa is resected.^18^ In our study, patients with defects involving 75%–89% of the esophageal circumference had an increased stricture rate as compared to <50% involvement, further highlighting that even sub-total circumferential resections carry a high risk of stenosis. The abrupt increase in stricture formation beyond the 75% threshold suggests that there may be a critical point at which the esophageal mucosa loses its ability to regenerate without significant cicatricial contraction.^17^ This finding is consistent with prior research indicating that defects larger than three-quarters of the circumference place patients at the highest risk of stricture formation, necessitating aggressive prophylactic interventions.^21^ For cases where large circumferential resections are unavoidable, adjunctive strategies such as prophylactic steroid therapy may be beneficial, although their efficacy remains to be clearly established in prospective trials.^22^ Additionally, procedural modifications, including a step-wise approach whereby one avoids complete or near-complete circumferential resections can be considered.

The differences in circumferential involvement between BE-related dysplasia and SCD provide further insight into stricture risk variation between these two groups. While the overall rate of strictures was higher in patients with BE compared to those with SCD, this may be partially explained by differences in lesion size and circumferential involvement. In our cohort, patients with BE dysplasia had a higher proportion of lesions involving ≥75% of the esophageal circumference compared to those with SCD. Specifically, 17.1% of BE patients had near-complete circumferential involvement (90%–100%), while only 5% of SCD patients had lesions of this extent.^23^ This aligns with prior observations that Barrett’s-associated lesions tend to be more extensive, particularly in the setting of long-segment BE, whereas squamous lesions are often smaller and more focal.^24^^,^^25^ Given the well-established correlation between circumferential defect size and stricture formation, the higher rate of extensive resections in BE patients likely contributed to this increased stricture risk.^25^ Additionally, lesion characteristics may influence resection dynamics and healing responses. These benchmarks frame our Western cohort; prevention remains particularly challenging for near-circumferential defects in Western circumferential ESD experience.^26^ Independent of therapy-related fibrosis, Barrett’s mucosa shows fibromuscular remodeling—duplicated/thickened muscularis mucosae and subepithelial fibrosis—reported in resection/EMR series.^27^^,^^28^ BE lesions are often associated with more significant submucosal fibrosis due to chronic gastroesophageal reflux disease (GERD)^29^ and prior resection or ablative therapies,^30^ which may complicate dissection and increase the likelihood of deeper mural injury. Nonetheless, the increased lesion size and circumferential involvement in BE-related dysplasia likely contributed to the higher stricture rate observed in this subgroup.

DMI was another significant risk factor associated with stricture formation.^31^ This finding emphasizes the role of meticulous tip control and technique to prevent unintended damage to deeper esophageal layers, which can exacerbate post-procedural inflammation and fibrosis.^32^ The development of strictures in patients with DMI is likely multifactorial. When the submucosal plane is disrupted beyond the intended resection depth, exposure of, or penetration through, the muscularis propria may trigger an excessive fibrotic response during the healing process.^17^^,^^33^ This can result in extensive collagen deposition and scar contracture, significantly increasing the risk of luminal narrowing.^17^^,^^33^ Additionally, deeper injury may impair normal re-epithelialization, prolonging the inflammatory phase of healing and further predisposing the affected segment to stricture formation.^17^ An alternative explanation is that the presence of significant fibrosis itself may contribute to the risk of DMI rather than DMI solely predisposing patients to strictures. Extensive submucosal fibrosis, often seen in cases of recurrent dysplasia,^34^ prior endoscopic interventions, or chronic inflammation, can make achieving a clean submucosal dissection plane technically challenging.^35^ In these cases, the difficulty in maintaining an appropriate resection depth may increase the likelihood of DMI. At the same time, the presence of DMI further exacerbates local inflammation and fibrosis, ultimately increasing the risk of stricture formation. This bidirectional relationship suggests that fibrosis and DMI may act synergistically, creating a cycle that predisposes to more aggressive post-ESD stricture formation. In our cohort, DMI occurred significantly more frequently in patients who later developed strictures. This suggests that tissue trauma during the procedure plays a crucial role in post-ESD outcomes. The increased prevalence of deep injury in the stricture group may reflect more challenging resections, such as those involving larger lesions, extensive circumferential involvement, or fibrotic, non-lifting lesions requiring aggressive dissection.^36–38^

Longer hospitalization duration was independently associated with stricture formation. Patients requiring extended hospital stays likely represent more complex cases, often characterized by larger resections, intraoperative complications, or increased post-procedural morbidity. Procedural complexity was likely a key factor, as patients who developed strictures had larger circumferential defects, which have been strongly linked to prolonged procedure times and increased risk of post-procedural complications.^16^ Additionally, intraoperative complications such as DMI were significantly more frequent in patients who developed strictures, which may have necessitated extended monitoring and supportive care. Overall perforation rates remain low post-ESD (2/108: 1.9%). However, post-procedural complications, particularly perforation, were more common in patients who later developed strictures. Perforation occurred in patients in the stricture group, whereas no perforations were reported in those without strictures (*p* = 0.011). These patients likely required prolonged observation, additional interventions, or delayed oral intake, further extending hospitalization. Another factor contributing to prolonged hospitalization and subsequent stricture formation may be the delayed initiation of prophylactic steroid therapy.^11^^,^^39^^,^^40^ While prolonged hospitalization was independently associated with stricture formation, this relationship may reflect underlying confounding factors rather than a direct causal link. It is likely that the extended length of stay serves as a proxy for unmeasured variables, such as aspects of procedural complexity, tissue healing dynamics, or patient comorbidities, which warrant further investigation.

Despite the high incidence of strictures in patients with large resection defects, our findings suggest that prophylactic strategies may have variable efficacy.^41^ Among patients with circumferential resection defects exceeding 70% (*n* = 56), those who received intraprocedural steroid injection with LTA (*n* = 37) had a stricture rate of 45.9% (17/37), with 43.2% (16/37) developing severe strictures. In contrast, among those who did not receive LTA (*n* = 19), the stricture rate was 21.1% (4/19), with severe strictures occurring in 15.8% (3/19). Although LTA is intended to reduce stricture formation, its apparent association with a higher stricture rate in this study likely reflects a selection bias, as LTA was preferentially administered to patients with the highest risk of stricture formation based on endoscopic judgment. Patients who received LTA may have had more extensive or deeper resections, predisposing them to a higher baseline risk of stricture development regardless of intervention. Additionally, the potential variability in steroid application techniques, timing, or dosing could also influence efficacy.^40^^,^^42^ Moreover, the absolute stricture rates in steroid-treated patients remained high, particularly for those with ≥75% circumferential defects, suggesting that steroids alone may be insufficient for extensive resections.^11^^,^^39^ Systemic steroid therapy demonstrated mixed outcomes. Patients discharged on prophylactic steroids exhibited differing stricture rates based on the regimen: prednisone (61.5%), oral budesonide (26.9%), and combination therapy (7.7%). While steroid use was marginally protective, its impact did not appear to be sufficient to fully prevent stenosis in high-risk patients.^41^ It is worth noting that prior studies evaluating steroid therapy for post-ESD stricture prevention have demonstrated benefit, but these studies often included larger patient cohorts.^11^^,^^43^^,^^44^

This study is limited by its retrospective design and single-center setting may limit generalizability to broader patient populations and different institutional practices. Second, the relatively small sample size, particularly in the subgroup of patients with squamous cell dysplasia, may have limited the statistical power to detect additional risk factors or subtle differences between groups. Additionally, while we identified independent predictors of stricture formation, the impact of specific prophylactic strategies remains unclear due to variability in their application and potential confounding factors. We also acknowledge that our predictor analysis is subject to confounding. Circumferential extent of resection is a dominant driver of stricture risk, and variables such as procedure time, hospitalization duration, and complications may represent downstream consequences of more extensive resections rather than truly independent risk factors. Although we attempted to adjust for collinearity in multivariable modeling, residual confounding likely remains, and the findings should be interpreted as complementary markers of procedural complexity rather than discrete causal predictors. Future prospective studies with larger, multicenter cohorts are necessary to validate our findings and assess the efficacy of multimodal preventive approaches, including early prophylactic stent placement with fixation, early and aggressive prophylactic dilation, biologic scaffolds, and novel anti-fibrotic agents.

In brief, our analysis supports moving toward a more structured, risk-stratified pathway: intra-procedurally, patients with (i) a mucosal defect ≥75% circumference or (ii) any DMI would be designated high-risk; for these patients, we propose closer follow-up endoscopy (2–4 weeks) and careful consideration of early intervention strategies. The potential role of pre-emptive dilation in asymptomatic patients remains uncertain and should be formally evaluated in prospective trials before it can be recommended in clinical practice.^10^^,^^45^

## CONCLUSION

The identification of key risk factors of stricture formation supports the need for tailored risk stratification to guide prophylactic and therapeutic strategies. Patients with extensive circumferential resections, DMI, or prolonged hospitalization represent high-risk groups that may benefit from early, aggressive intervention. While steroids remain a cornerstone of prophylactic therapy, their efficacy appears limited in extensive resections, necessitating the exploration of adjunctive measures.^46^ Further research is needed to refine and optimize prophylactic strategies for high-risk patients undergoing esophageal ESD.

## Supplementary Material

Supplementary_Data_doaf096

## ABBREVIATIONS

APC, argon plasma coagulation; BE, Barrett’s esophagus; CAD, coronary artery disease; CHF, congestive heart failure; CI, confidence interval; COPD, chronic obstructive pulmonary disease; DLD, dyslipidemia; EMR, endoscopic mucosal resection; ESD, endoscopic submucosal dissection; EUS, endoscopic ultrasound; GERD, gastroesophageal reflux disease; GI, gastrointestinal; HTN, hypertension; IBD, inflammatory bowel disease; IQR, interquartile range; NBI, narrow-band imaging; OR, odds ratio; PPI, proton pump inhibitor; PAD, peripheral artery disease; RFA, radiofrequency ablation; SCD, squamous cell dysplasia; UC, ulcerative colitis; VIF, variance inflation factor; WLE, white-light endoscopy.

## Notes

Specific author contributions: Kareem Khalaf conceived the study idea, conducted the analysis, collected data, and contributed to writing the manuscript. Huaqi Li performed the data analysis, wrote the results section, and drafted the tables. Youstina Hanna and Tomoyuki Nishimura were responsible for data collection and manuscript editing. Natalia Causada Calo, Gary R. May, and Christopher Wayne Teshima reviewed, edited, and approved the final version. Jeffrey D. Mosko and Christopher Wayne Teshima organized the project and served as Principal Investigators (PI). All authors reviewed, edited and approved the final version of the manuscript. Kareem Khalaf (Conceptualization, Data curation, Formal analysis, Investigation, Methodology, Project administration, Software, Supervision, Validation, Visualization, Writing—original draft), Youstina Hanna (Data curation, Writing—review & editing), Tomoyuki Nishimura (Data curation, Writing—review & editing), Huaqi Li (Formal analysis, Methodology, Writing—review & editing), Natalia Causada Calo (Writing—review & editing), Christopher Wayne Teshima (Conceptualization, Writing—review & editing), Jeffrey D. Mosko (Conceptualization, Methodology, Project administration, Supervision, Validation, Writing—review & editing), and Gary R. May (Writing—review & editing)

Financial support: None was required for the completion of the study.
